# Carrageenan Edible Coating Application Prolongs Cavendish Banana Shelf Life

**DOI:** 10.1155/2020/8861610

**Published:** 2020-08-28

**Authors:** Fenny Martha Dwivany, Ayesha Nilam Aprilyandi, Veinardi Suendo, Nisrina Sukriandi

**Affiliations:** ^1^School of Life Science and Technology, Institut Teknologi Bandung, Bandung 40132, Indonesia; ^2^Research Center for Nanosciences and Nanotechnology, Institut Teknologi Bandung, Bandung 40132, Indonesia; ^3^National Agency of Drug and Food Control Republic of Indonesia, Jakarta 10560, Indonesia; ^4^Chemistry Department, Faculty of Mathematics and Natural Sciences, Institut Teknologi Bandung, Bandung 40132, Indonesia

## Abstract

Banana is very important for both food and economic securities in many tropical and subtropical countries, because of its nutritional values. However, banana fruit is a climacteric fruit which has short shelf life, so an alternative method to delay its ripening is needed. Our group has used carrageenan as an edible coating to delay banana fruit ripening. In this study, the effect of different concentrations of carrageenan and storage temperatures on Cavendish banana shelf life and fruit quality was evaluated. The fruits were treated with 0.5%, 1.0%, and 1.5% carrageenan and stored at two different temperatures, 26°C and 20°C. Carrageenan functional groups in banana peel samples as well as changes in surface structure of banana peel, color, weight loss, pulp to peel ratio, total soluble solid, and levels of *MaACS1* and *MaACO1* gene expression were analyzed. Result showed that the optimum condition to extend shelf life and maintain fruit quality was by treating the banana fruits with 1.5% carrageenan and storing them at a cool temperature (20°C). In addition, the result obtained from this study suggested that carrageenan can be used as edible coating to extend the shelf life of banana fruits (*Musa acuminata* AAA group).

## 1. Introduction

Banana is one of the most popular fruits that are in great demand. The Cavendish banana cultivar is commonly consumed as a quick dessert. Its high nutrient content makes it attractive to be consumed daily. Banana fruit is a climacteric fruit that will quickly ripen after harvest [1]. The ripening process of climacteric plants is accompanied by an increase in respiration and ethylene production, a hormone that is involved in fruit ripening [2]. The biosynthesis of ethylene is regulated by two important genes, *Musa acuminata* aminocyclopropane-1-carboxylic acid synthase (*MaACS1*) and *Musa acuminata* aminocyclopropanecarboxylate oxidase (*MaACO1*). These genes encode ACC (1 aminocyclopropane-1-carboxylic acid) synthase (ACS) and ACC oxidase (ACO), respectively, and catalyze the synthesis of ethylene from its precursor *S-*AdoMet (*S-*adenosyl-methionine/SAM) and ACC (1 aminocyclopropane-1-carboxylic acid) [3]. This process is also known to be affected by O_2_ and CO_2_ during fruit respiration [4]. The presence of ethylene surrounding the fruit will activate genes for the process of fruit ripening and will speed up the fruit senescence response. These genes induce physiological processes in fruits such as ethylene biosynthesis [5], respiration, starch metabolism, and degradation of the cell wall [6].

One alternative method to increase the fruit shelf life is by using edible coating. Edible coating could prevent O_2_ penetration into the fruit and inhibit microbial growth [7]. The use of edible and biodegradable coatings has been encouraged to extend shelf life, improve food quality, and reduce packaging waste [8]. The use of these biodegradable materials could to some extent help solve the waste problem [9]. Our previous study showed that chitosan as edible coating for banana fruit could slow down ripening of the banana [10, 11].

Carrageenan, extracted from the red alga *Eucheuma cottonii* (*Rhodophyceae*), had been reported to be used as edible packaging material for fruits [12–14]. The use of carrageenan as edible coating had been done on home industry as well as large industry scale. The carrageenan edible coating could become a selective permeable membrane for O_2_ and CO_2_ gas [15]. This polysaccharide-based membrane could modify the internal atmosphere of the fruit and thus extend its shelf life [16]. Carrageenan had been used as edible coating for fresh-cut banana [12]. However, the effect of this coating on intact fruits and gene expression related to ethylene biosynthesis had not been reported yet. The objective of this study was to apply carrageenan edible coating on Cavendish banana fruits and store them at different temperatures to maintain quality and extend shelf life. Physical and physiological analyses were performed. Expression of genes involved in ethylene biosynthesis (*MaACO1* and *MaACS1*) was analyzed with quantitative polymerase chain reaction (qPCR). The expression profile of these two genes could become a molecular marker for carrageenan treatment in banana fruit ripening.

## 2. Materials and Methods

### 2.1. Materials

The Cavendish Ambon banana cultivar (AAA) was obtained from PT Sewu Segar Indonesia (Tangerang, Indonesia). The green matured bananas in this study had been exposed to 100 ppm ethylene at 14°C for 24 hours. The criteria for the experimental banana fruits were absence of physical defects on the skin or the pulp. The experimental fruits all had the same physiological age, color, and size. The banana fruits were separated into fingers from the side and randomly grouped into four treatments: coated with 0.5% (*w*/*v*), 1% (*w*/*v*), and 1.5% (*w*/*v*) carrageenan or not coated combined with storage either at room temperature (26 ± 1°C) or in an air-conditioned room (20°C). Each treatment had three replicates.

### 2.2. Preparation of the Carrageenan Solution

The carrageenan solution was prepared according to the method of edible coating with modification [15]. Distilled water (1.5 L) was heated on a magnetic hot plate while being stirred until it reached 80°C; then, carboxymethyl cellulose (CMC) was added to dissolve until it reached 1% (*w*/*v*). Carrageenan powder was added to the solution followed by glycerol to a final concentration of 0.5% (*v*/*v*). The solution was stirred at 80°C for 30 more minutes until homogeneous, then stored at room temperature until it reached 50°C.

### 2.3. Coating Banana with Carrageenan Solution

Coating was performed by dipping the fruits in 0.5%, 1.0%, and 1.5% carrageenan solutions for around 30 seconds at 50°C. After dipping, the bananas were hanged to air-dry at room temperature. The bananas were then placed in storage racks at either 26± 1°C or 20°C. As control, banana fruits that had not been coated with carrageenan were stored at 26 ± 1°C and 20°C.

### 2.4. Characterization of Carrageenan-Coated Peel

The presence of 1.5% carrageenan on the surface of the banana fruit peel was confirmed by attenuated total reflection (ATR) spectroscopy using an ALPHA FTIR Spectrometer (Bruker, Billerica, MA, USA).

### 2.5. Scanning Electron Microscopy (SEM)

Square pieces (1 cm × 1 cm) of carrageenan-coated and uncoated peels of 5 mm thick were freeze-dried for seven hours and analyzed using SEM (JSM-6510LA, JEOL Ltd., Tokyo, Japan) [10].

### 2.6. Physical and Biochemical Analysis of Banana Fruit Ripeness

Physical and biochemical analyses during fruit ripening [17] were performed on days 1, 3, 5, 7, 9, 11, and 13. Observations included change in peel color, measurement of peel to pulp ratio, starch percentage analysis of the pulp using iodine test, and measurement of the total soluble solid (TSS) content using a refractometer (Atago Co. Ltd., Tokyo, Japan). TSS content was measured according to the method used in a previous study [18], and the results were expressed as degree Brix (°Brix).

Conversion of starch into sugar during ripening was assessed by measuring the starch percentage of the pulp. The starch-iodine staining solution consisted of 1% potassium iodide and 0.25% iodine. The banana was cut transversely about 2-3 cm thick at midpoint, and the peel was separated from the pulp. The surface of the cut banana was dipped 5 mm deep in the starch-iodine staining solution for 5 seconds. The starch pattern of each fruit was analyzed by comparing it to a starch iodine staining chart for bananas [19].

Statistical analyses were performed at all the results, in order to determine which treatment groups had significant differences of TSS, weight loss, and pulp to peel ratio, compared to another groups. Tests of normal distribution were conducted using Kolmogorov-Smirnov test. Data with normal distribution were then analyzed using the ANOVA test and further analyzed using the Tukey post hoc test. Data that were not normally distributed were analyzed using Kruskal-Wallis test and further analyzed using Mann-Whitney *U* test.

### 2.7. Gene Expression Analysis

Total RNA was isolated from the pulp of the banana fruit following the protocol used in the previous study [20]. RNA isolation was performed for samples of days 1, 7, and 13. Total RNA was extracted from ground banana pulp using the extraction buffer (2% cetyltrimethylammonium bromide; 2% polyvinylpyrrolidone; 100 mM trishydroxymethyl aminomethane-HCl pH 8; 25 mM ethylenediaminetetraacetic acid; 2 M NaCl; and 2% 2-mercaptoethanol) and recovered using lithium chloride (LiCl). Firstly, the middle part of banana fruit was cut and quenched by liquid nitrogen. Two grams of sample was ground to powder using a mortar with liquid nitrogen. The fine powder was divided evenly to 4 of 1.5 mL micro tubes filled with 750 *μ*L preheated extraction buffers at 65°C. In addition, 30 *μ*L *β*-mercaptoethanol was added to the buffer immediately before use.

The sample and extraction buffer were homogenized in the micro tube using vortex for 1 minute. Then, the mixed solution was incubated at 65°C for 15 minutes with vortex for 1 minute every 5-minute interval. An equal volume (750 *μ*L) of chloroform, isoamylalcohol (24 : 1), was added after the solution reached room temperature. Subsequently, the solution was vortexed for 10 minutes and centrifuged at 8000 rpm for 10 minutes at 4°C. The aqueous phase (500 *μ*L) was transferred into a new micro tube using a micropipette. Then, the process from adding chloroform, isoamylalcohol (24 : 1), until taking the aqueous phase was repeated. 500 *μ*L of the supernatant was obtained, and 1/3 volume (167 *μ*L) of 7.5 M lithium chloride (LiCl) was added. The solution was homogenized slowly by inverting the tube 10 times and then incubated for 16-18 hours at 4°C. The incubated solution was centrifuged at 8000 rpm for 30 minutes at 4°C. The supernatant was discarded, and the obtained pellet was dissolved in 500 *μ*L of diethylpyrocarbonate- (DEPC-) treated water, and 1/5 volume (63.3 *μ*L) of 3 M sodium acetate (pH 5.2) and 2 volumes (1 mL) of 100% ethanol were added and slowly homogenized by inverting the tube. The solution was incubated at -20°C for 2 hours. The incubated solution was centrifuged at 8000 rpm for 10 minutes at 4°C. Then, the supernatant was removed, and the obtained RNA pellet was washed with 70% ethanol and centrifuged at 8000 rpm for 5 minutes at 4°C. The supernatant was subsequently discarded, and the RNA sample was dried by turning the micro tube over on dry tissue for approximately 25 minutes. The RNA sample was resuspended in 30 *μ*L DEPC-treated water and stored at -80°C until use. Concentration and purity of the RNA sample was measured using NanoDrop™ 2000C (Thermo Fisher Scientific, Waltham, MA, USA), at wavelengths of 260, 280, 230, and 320 nm. Contaminating genomic DNA was removed from the total RNA by digestion using DNAse I kit (catalog no. 89836, Thermo Fisher Scientific™, Waltham, MA, USA). The purified total RNA was used as template for cDNA synthesis using iScript™ cDNA synthesis kit (catalog no. 1708890, Bio-Rad, Philadelphia, PA, USA).

Quantification of the level of mRNA was performed using CFX96 Touch Real-Time PCR Detection System (Bio-Rad, Philadelphia, PA, USA) that was connected to IQ™5 Real-Time PCR Detection Systems (Bio-Rad, Philadelphia, PA, USA). Primers for *MaAC*S1 and *MaACO1* genes, as well as *MaGAPDH* as housekeeping gene, were the ones used in previous studies [10, 21, 22]: MaACS1_F 5′-CCGAGACTGGATGAAGAAGAA-3′; MaACS1_R 5′-GTCTGGGTCAAATCTGGCTC-3′; MaACO1_F 5′-CGAGATGCTTGCGAGAAATGG-3′; MaACO1_R 5′-TGCAGCAAATTCCTTCATCGC-3′; MaGAPDH_F 5′-TCAACGACCCCTTCATCAC-3′; and MaGAPDH_R 5′-AGCAGCCTTGTCCTTGTCA-3′.

## 3. Results

### 3.1. Characterization of the Carrageenan Functional Groups

**Figure 1:**
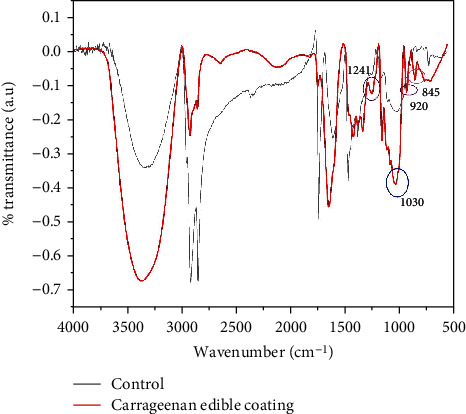
FTIR spectra of samples from banana peel of the control and carrageenan- (1.5%) coated banana peel. Functional groups present in carrageenan within wavenumbers 1500-500 cm^−1^ are circled. The picture shows the FTIR spectra in wavenumber region 4000-500 cm^−1^.

FTIR analysis was performed to confirm the coating of banana peel with 1.5% carrageenan. Samples of banana peel coated with carrageenan as well as uncoated banana peel as control were analyzed with FTIR. Changes in transmittance intensity in the spectrum are shown in (Figure 1).

The FTIR spectrum of the coated banana samples showed several specific peaks for functional groups present in carrageenan. There were very strong peaks at wavenumber 1241 cm^−1^ for the S=O bond of sulfate ester, 1030 cm^−1^ for glycoside bonds, a typical peak for galactose-4-sulfate at 845 cm^−1^, and a typical peak for 3,6-anhydro-D-galactose at 925 cm^−1^ [23].

### 3.2. Characterization of Banana Peel with Scanning Electron Microscopy (SEM)

**Figure 2:**
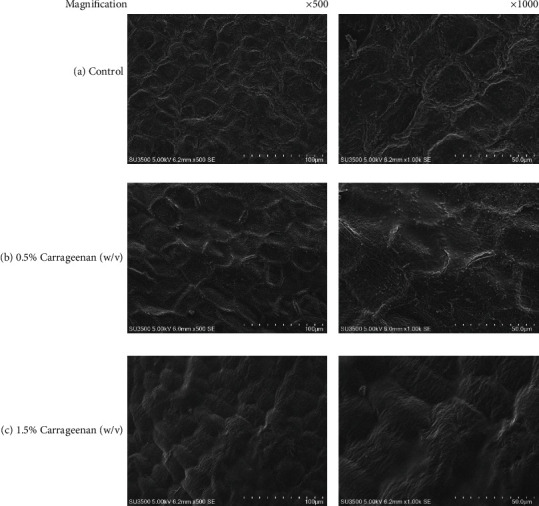
Scanning electron micrograph of banana peel: (a) control (without edible coating), (b) 0.5% (*w*/*v*) carrageenan edible coating, and (c) 1.5% (*w*/*v*) carrageenan edible coating.

Scanning Electron Microscopy (SEM) was performed on the banana peels to view the morphology of the banana peels with their carrageenan edible coatings. Samples of banana peel without treatment and banana peel treated with 0.5% and 1.5% carrageenan solutions were analyzed. The electron micrographs with 500x and 1000x magnifications are shown in (Figure 2).

Banana peels coated with carrageenan (Figures 2(b), and 2(c)) had a smoother texture compared to the control (a). The higher carrageenan concentration used resulted in thicker coatings. The results showed that coating with 0.5% carrageenan did not delay fruit ripening. Treatment with 0.5% carrageenan could not effectively coat the surface of the peel because the gel that was produced was thinner and the coating was uneven as shown in the electron micrograph.

### 3.3. Changes in Banana Peel Color and Starch Percentage

**Figure 3:**
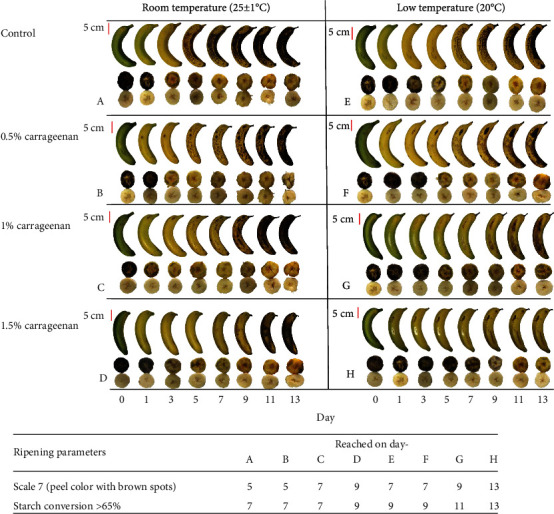
Change in peel color and starch conversion analysis by starch iodine stain from day 0 to day 13 in banana from different treatments. Treatment groups are coded by alphabets A to H. Data shown was one of three biological replicates.

Changes in the color of banana peel and starch percentage of the pulp during 13 days of storage are shown in (Figure 3).

There were differences in the changes of the banana peel color and the starch percentage of the pulp between the treatments. Banana that did not get any coating and stored at low temperature took two days longer to ripen compared to the control but had the same starch conversion rate with complete conversion on day 7. Within treatments that were stored at room temperature, the 1.5% carrageenan treatment took the longest time to ripen, with a shelf life two days longer than the control. Within treatments stored at low temperature, the one treated with 1.5% carrageenan took the longest time to ripen and the shelf life was six days longer than the control.

### 3.4. Change in the Total Soluble Solid

**Figure 4:**
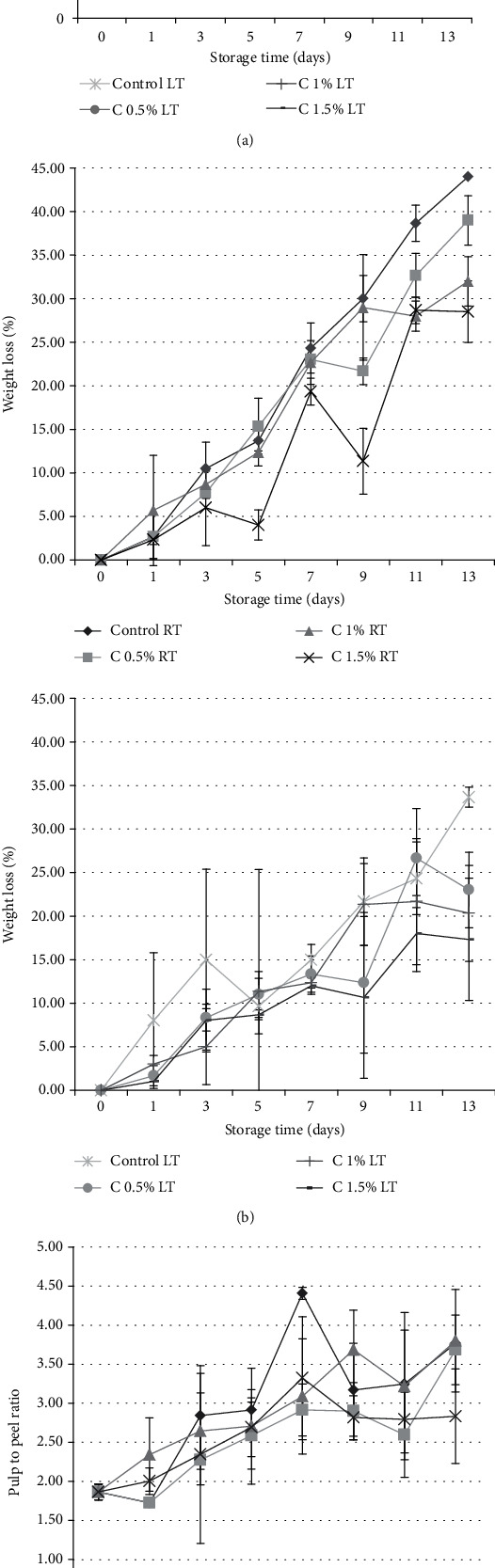
(a) Changes in total soluble solid (TSS) in the pulp of fruits from eight different treatments during ripening. Statistical analyses showed that the treatment groups C 0.5% LT and C 1.5% LT had significantly lower TSS, compared to its control group (control LT). Meanwhile, there were no significant differences in TSS of all treatment groups at room temperature (RT). (b) Changes in weight loss of fruits from eight different treatments during ripening. Statistical analyses showed that the treatment groups C 1% LT and C 1.5% LT had significantly lower weight loss, compared to its control group (Control LT). Meanwhile, there were no significant differences in weight loss of all treatment groups at room temperature (RT). (c) Changes in pulp to peel ratio of eight different treatments during ripening. Statistical analyses using ANOVA showed that the treatment group C 0.5% RT had significantly lower pulp to peel ratio, compared to its control group (control RT). Meanwhile, there were no significant differences in pulp to peel ratio of all treatment groups at low temperature (LT). All data means of three biological replicates.

Results of the total soluble solid (TSS) analysis in (Figure 4) showed similar patterns for all the treatments.

In the control group, there was a high increase in TSS at the beginning followed by a lesser increase up till day 11; then, the TSS decreased. The pattern of TSS changed on 0.5% and 0.1% carrageenan edible coating where the peak TSS was reached earlier followed by a decrease in TSS. The lowest TSS was obtained from 1.5% carrageenan edible coating stored at low temperature.

### 3.5. Weight Loss of the Banana Fruit

Weight loss during ripening of the banana fruits from each treatment is shown in (Figure 4(b)).

The level of weight loss at each observation point varied between each treatment, with a tendency to increase from the beginning to the end of the treatment. The longer the storage time of the fruit, the higher the weight loss. In all the treatments, there were occasional decrease and increase of weight loss at certain observation points. The weight loss value was obtained from the average of three replicates that started with banana fruits with different initial weight, and this could cause variations in the data measurement.

### 3.6. Banana Fruit Pulp to Peel Ratio

The pulp to peel ratio of banana fruits from each treatment is shown in (Figure 4(c)).

The general pattern of the pulp to peel ratio tended to increase in each treatment, with increasing and decreasing ratios at a few observation points. The change in the values of the ratios can originate from the fact that the average of the three replicates comes from bananas that do not have the same initial weight which caused variations in the data measurement. Based on the average pulp to peel ratio, it could be concluded that treatment with 1.5% (*w*/*v*) carrageenan edible coating stored at low temperature had the lowest pulp to peel ratio compared to the other treatments.

### 3.7. Analysis of *MaACS1* and *MaACO1* Expression

Analysis of gene expression was performed on two fruit samples, the optimum treatment (1.5% carrageenan edible coating combined with storage at low temperature) and control at room temperature (uncoated and storage at room temperature), on days 1, 7, and 13. Two genes involved in the biosynthesis of ethylene, *MaACS1* and *MaACO1*, were analyzed. *MaGAPDH* was used as the reference gene for normalization of data.

**Figure 5:**
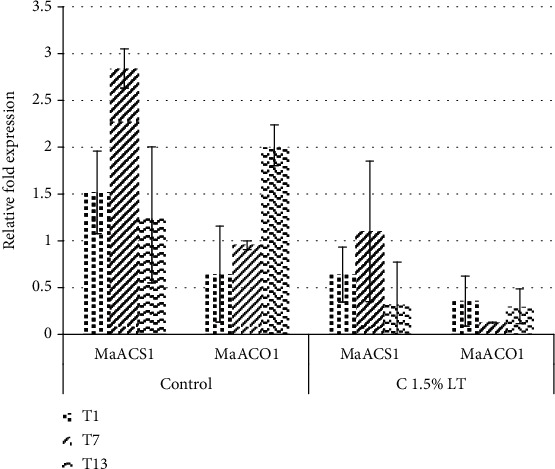
Relative expression of *MaACS1* and *MaACO1* genes in the pulp during ripening of banana fruits from the control (no edible coating, storage at room temperature 26°C) and optimum treatment (1.5% carrageenan edible coating and storage at low temperature 20°C). Data means of three biological replicates.

Results of the qPCR experiment showed different profiles of relative *MaACS1* and *MaACO1* gene expression in the pulp of the control and the optimum treatment (Figure 5).

Relative *MaACS1* gene expression in the control sample showed an increase in expression, with a peak on day 7, followed by a decrease toward the end of the observation period while the optimum treatment showed lower *MaACS1*gene expression level. On the other hand, the relative *MaACO1* gene expression had different patterns between the control and the optimum treatment.

## 4. Discussion

### 4.1. Carrageenan Edible Coating in Combination with Low Temperature Was Effective to Prolong Banana Shelf Life

These results were in agreement with the results obtained from edible coating with chitosan as reported in a previous study [10]. Banana fruit coated with 1.15% and 1.25% chitosan (CS) had longer shelf life compared to banana coated with chitosan nanoparticle (CN). This might be due to the thicker coating as seen on the SEM electron micrograph of the peel surface. The surfaces of banana peels treated with 1.15% and 1.25% CS were completely covered with chitosan coating. However, banana treated with a higher concentration of chitosan did not ripen properly [10]. A thick chitosan coating could have hindered gas diffusion [24] that could result in the generation of heat and anaerobic condition, leading to banana ethanol production [25].

The results also showed that lower temperature could also delay ripening compared to room temperature. Ethylene biosynthesis was affected by temperature, increasing with the increase in ripening temperature until a certain point [26]. In this study, lower temperature could result in lower metabolic processes as shown by slower rate of the peel color changes and amylum conversion in the pulp. These results confirmed the results in a previous study [11] that bananas stored at 20°C had better physical characteristics than bananas stored at room temperature. Therefore, it could be concluded that based on the slower change in the color of the peel and in amylum conversion, the most optimal treatment for the banana fruit was with 1.5% (*w*/*v*) carrageenan edible coating and stored at low temperature (20°C).

It had been known previously that during banana fruit ripening, there was a gradual conversion of amylum into simple sugars. That is why the more advanced stage of ripening had higher TSS because the starch had been converted into sugar [17]. The same pattern of TSS was obtained in a previous study [10] where there was an increase in TSS at the beginning of the ripening process followed by a decrease. Besides that, treatment with chitosan edible coating was reported to decrease TSS compared to control. From this research, it could be concluded that treatment with 1.5% carrageenan edible coating combined with storage at low temperature could slow down the ripening process as shown by the lowest average value of TSS compared to all the other treatments.

The average weight loss of the low temperature (9.46%) treatment was lower than the room temperature one (20.48%). This could be caused by the slower metabolism at low temperature which would then decrease the respiration and transpiration rate of the fruit, and thus, there would be less water loss [27]. Variation in the concentration of carrageenan edible coating also resulted in different average weight loss of the banana fruit. Higher concentration of carrageenan edible coating resulted in lower banana fruit weight loss. The edible coating could function as barrier of water vapor for the banana fruit [28]. As mentioned above, the use of higher concentration of carrageenan produced thicker edible coating on the peel of the fruit as shown in the electron micrograph in Figure 2. Edible coating such as chitosan could become a barrier and reduce the O_2_ supply to the banana fruit [29]. High concentration of CO_2_ and low concentration of O_2_ could inhibit degradation of chlorophyll in the banana peel [30] and ethylene production, therefore delaying fruit ripening [30]. Based on the average weight loss of the banana fruit, it could be concluded that treatment with 1.5% (*w*/*v*) carrageenan edible coating stored at low temperature could optimally reduce water transpiration from the banana fruit compared to the other treatments.

Treatment with carrageenan edible coating and low temperature could decrease the pulp to peel ratio of banana fruit. The edible coating could become a shield for the banana fruit. Storage of fruits at low temperature could decrease the rate of respiration and transpiration of the fruit, thus preserving the water content of the pulp and peel. During fruit ripening, the pulp to peel ratio of the banana fruit would consistently increase [17]. The presence of the hormone ethylene during ripening causes the conversion of amylum into simple sugars. The increase in sugar concentration in the pulp during ripening process would cause osmosis of water from the peel to the pulp of the fruit [27]. Besides that, the process of cell respiration produced water in the pulp of the fruit and this water could not be immediately released to the atmosphere, as was the case for the peel of the fruit; therefore, accumulation of water in the pulp occurred. Therefore, the pulp to peel ratio would increase during ripening [17]. Based on the changes in peel color, starch percentage, TSS, percent weight loss, and pulp to peel ratio, treatment with 1.5% carrageenan edible coating combined with storage at low temperature (20°C) was the best treatment to optimally delay food ripening and preserve the taste and physical quality of the banana fruit. This treatment could maintain shelf life six days longer than the control. For this reason, next gene expression analyses were performed on this treatment.

### 4.2. Carrageenan Edible Coating Affected Both *MaACS1* and *MaACO1* Gene Expressions

This was in agreement with the results of previous studies [5, 10, 21], where the pattern of gene expression showed an increase to a certain point in time followed by a decrease toward the end of the ripening period. On the other hand, the increase in *MaACS1* gene expression in the optimum treatment tended to be lower. In contrast to *MaACS1* gene expression, the pattern of *MaACO1* gene expression of the control was different from the optimum treatment. In the control sample, the expression of *MaACO1* gene increased from the beginning to the end of the observation period on day 13, while in the optimum treatment, expression decreased from the beginning followed by increase and decrease to the end of the observation period. The level of *MaACO1* gene expression of the treated sample was lower than that of the control.

In climacteric fruits such as banana, there is a significant increase in respiration and production of endogenous ethylene at the beginning of ripening, concomitant with the increase in *MaACS1* and *MaACO1* expression. The high increase in *MaACS1* in fruit ripening indicated a pattern of specific ethylene interaction during the ripening process of climacteric plants. Ethylene production would increase then decrease in a relatively short period of time at the beginning of the increase in respiration that coincided with the beginning of fruit ripening, which was observed through the expression of ripening genes [5]. The high increase in ethylene production could occur because of the high expression of *MaACS1* gene that would increase ACC synthase synthesis. High ACC synthase would result in the increase in ACC synthesis which would cause an increase of ethylene synthesis by the fruit.

*MaACO1* gene expression tended to be lower than the expression of *MaACS1* gene. This was in agreement with a previous study [30] that stated that based on RT-PCR and immunoblotting, MaACS1 protein production was higher than MaACO1. This was probably due to the increasing *MaACS1* expression during ripening; meanwhile, expression of the *MaACO1* gene since the preclimacteric period was maintained until the end of ripening [31, 32]. ACC oxidase enzyme coded by the *MaACO1* gene was affected by the presence of O_2_ in the cell. O_2_ in the cell would help ACC oxidase convert ACC into ethylene. ACO enzyme was reported to be the limiting factor in ethylene biosynthesis [33]; therefore, even though *MaACS1* gene expression increased during the experiment, *MaACO1* expression remained low.

Carrageenan edible coating affected both gene expressions probably due to the limited O_2_ concentration in the cell as the consequence of the presence of a gas barrier. As mentioned above, the expression of *MaACO1* gene decreased from the beginning due to carrageenan coating. Previous studies using carrageenan as edible coating on strawberries had reported that it affected O_2_ permeability [15]. In the final reaction of ethylene biosynthesis, ACC oxidase (formerly ethylene forming enzyme (EFE)) catalyzed the reaction between ACC and O_2_ to produce ethylene [34]. The kinetics of this reaction indicated that it followed an ordered binding mechanism, where the enzyme first binds to O_2_ and then to ACC [35]. However, the expression of the *MaACO1* gene increased at the end of ripening which might due to ethylene accumulation that results in autocatalytic reaction and accumulation of ethylene due to a gas barrier. It has been reported that *LeACO1* gene expression may be enhanced during autocatalytic ethylene production [36].

In conclusion, treatment with 1.5% carrageenan edible coating and storage at low temperature (20°C) could lengthen the shelf life for six days compared to the control. Treatment of banana fruit with carrageenan edible coating and storage at room temperature could lengthen the shelf life for two days compared to the control, while storage at low temperature without edible coating could also lengthen the shelf life for two days compared to the control. Not only was it optimum for shelf life, treatment with 1.5% carrageenan edible coating and storage at low temperature (20°C) was also optimum for its effect on the expression of genes related to ethylene biosynthesis, *MaACS1* and *MaACO1*. Results from this study indicated that carrageenan edible coating has the potential to be used as an alternative material to lengthen the shelf life of Cavendish banana fruit.

## Data Availability

All data generated or analyzed during this study are included in this published article.
